# Examining the Dietary Diversity of Children in Niger

**DOI:** 10.3390/nu13092961

**Published:** 2021-08-26

**Authors:** Nafissatou Cisse Egbuonye, Ariun Ishdorj, E.L.J. McKyer, Rahma Mkuu

**Affiliations:** 1 Black Hawk County Health Department, Waterloo, IA 50703, USA; 2 Department of Agricultural Economics, Texas A&M University, College Station, TX 77843, USA; aishdorj@tamu.edu; 3 Department of Health Promotion and Community Health Sciences, School of Public Health, Texas A&M University, College Station, TX 77843, USA; mckyer@sph.tamhsc.edu; 4 Health Outcomes & Biomedical Informatics, College of Medicine, University of Florida, Gainesville, FL 32610, USA; rmkuu@ufl.edu

**Keywords:** women empowerment, rural, Niger, children, dietary diversity

## Abstract

Malnutrition is a major public health concern in Niger. The stunting rate in children in Niger is over 50%, one of the highest in the world. The purpose of this cross-sectional study was to examine children’s dietary diversity (CDD) and the maternal factors that impact CDD. A total of 1265 mother–child pairs were analyzed. Descriptive analysis was conducted to present maternal and child characteristics. To compare the mean scores of CDD in relation to the region, an independent sample *t*-test was conducted. A one-way ANOVA test was conducted to evaluate the CDD score by different age groups. A linear regression model was estimated to identify household, maternal and child factors that affect the CDD score. Our results indicate that most of the participants of our survey resided in rural areas and the majority (80.7%) of the mothers had no education. Factors such as region, children’s age, woman’s empowerment, vitamin A intake and wealth index were significant predictors of CDD (*p* < 0.05). The children residing in rural areas were more likely to have lower CDD scores (*p* < 0.05) than the children in urban areas, therefore becoming more susceptible to malnutrition.

## 1. Introduction

In Niger, approximately 10% of children aged 5 years or younger suffer from acute malnutrition, while 44% suffer from chronic malnutrition [1]. Malnutrition has a significant impact on infant morbidity and mortality outcomes and is the leading contributor to childhood stunting [2], which affects 48% of children 0–5 years of age in Niger [3]. The prevalence of malnutrition in Niger varies by region, with rural regions such as Diffa and Maradi and the urban region of Zinder experiencing the highest rates of malnutrition [4]. These alarming rates indicate that the majority of households in Niger are affected by malnutrition as a large proportion of the population resides in Diffa, Maradi and Zinder [5]. The prevalence of malnourishment is high due to a multitude of factors. Water is scarce because the country is landlocked in the Sahel, a region characterized by low amounts of rainfall annually [1]. In addition, inadequate agricultural production and national security constraints limit the availability of food [6]. Furthermore, Niger has one of the highest rates of population growth in the world: each year, the population grows by 4%, therefore contributing to food scarcity [1,7].

Among young children in developing nations, consuming foods that are diverse has been demonstrated to result in adequate nutrients intake needed for children’s healthy growth and development [8,9,10,11]. Thus, in 2007, the World Health Organization (WHO) introduced a Children’s Dietary Diversity (CDD) score, which is a dietary diversity measurement for children 6–23 months of age and is calculated as a sum of scores across the different food groups [12]. The consumption of foods from four or more food groups is specified as a nutritious diet for children [12]. In Sub-Saharan Africa (SSA), the majority of children do not meet this minimum diversity requirement [13,14,15]. In most households, the starchy staples food group is the most commonly consumed food group [16]. The dietary intake of infants and young children in SSA is largely composed of grain porridges, with low or no intake of vegetables and meat [3,17].

Similar to other countries in SSA, children in Niger have limited dietary diversity. Studies have found that children aged 0–5 years consume mostly grain porridges in the morning and mashed millet or sorghum accompanied by a vegetable-based sauce in the evening [18,19,20,21]. Vegetable-based sauces tend to be limited in micronutrients [18,21]. Moreover, the 2012 Demographic Health Survey for Niger yielded data demonstrating high difference in grain consumption between the breastfed and non-breastfed children (47.8% of the breastfed children vs. 75.2% of the non-breastfed children) [20,22]. In 2014, only 10% of the children 6–23 months of age met the minimum dietary diversity recommendations specified by the WHO [4].

According to the behavioral ecological model (BEM), malnutrition is a multifactorial trait caused by individual, household and environmental characteristics. The BEM incorporates learning theory with an ecological framework [23,24]. Under the BEM, behavior is conceptualized as a developmental process that is illuminated by and can be controlled through physical and social contingencies [23,24]. These physical and social contingencies can include reinforcers and other motivators of behavior [23,24]. According to this theoretical framework, a chain of these interacting contingencies can shape an individual’s behavior. This chain ranges from broad contingencies at the societal level to more physical and social contingencies at the individual level. It can be argued that dietary diversity can influence the child’s health outcomes as reflected by positive child anthropometric growth; however, different factors can also contribute to this outcome. Factors such as housing, socioeconomic status and access to medical care can all impact one’s health status [25]. Therefore, when examining causes of malnutrition in children, it is critical to not only study children’s eating behavior, but also examine maternal and environmental factors that can impact their dietary diversity and food intake. Limited empirical studies have been conducted that examine CDD of children in Niger. Hence, the purpose of this study was to (1) examine CDD of children 6–23 months of age in Niger, (2) determine whether differences in CDD exist between the children 6–23 months of age residing in urban areas versus those in rural areas and (3) determine maternal factors that affect CDD.

## 2. Materials and Methods

### 2.1. Data Source and Participants

This study used cross-sectional data from the 2012 Demographic and Health Survey (DHS) for Niger [26]. DHS is a publicly available nationally representative survey data that provides detailed information on a variety of variables relevant to a population’s health [26,27,28]. The survey has been widely used and is recognized as a reliable tool to provide nationally representative understanding of population health trends in developing nations. The data used the standard household questionnaire which was based on the model questionnaires [26,27,28]. Bearing in mind the needs of each specific country, DHS program staff work closely with local partners on the adaptation of the questionnaires. The survey is then translated to the main languages spoken in the country and tested in the field prior to its adoption [27]. In the case of Niger, the survey was translated, and the participants were interviewed using the main languages spoken in Niger (Hausa, Djerma) [26].

A two-stage cluster sampling method was utilized in eight regions (Agadez, Diffa, Dosso, Maradi, Tahoua, Tillaberi, Zinder and Niamey) in Niger to identify eligible households (i.e., those with at least one woman of reproductive age of 15–49 years), from which households were randomly selected. At the household level, eligible women were randomly selected and interviewed face-to-face to complete the questionnaires. The information on women’s characteristics and health behavior and the information on their children were collected.

The total sample size in the 2012 Niger DHS dataset was 11,160 women aged 15–49 years [26]. The WHO recommends exclusive breastfeeding for infants from birth to 6 months of age [14]; therefore, for the present study, we narrowed our sample to include the women with children aged 6–23 months. After removing missing data, the final study sample consisted of 1265 mother–child pairs.

### 2.2. Measures

#### 2.2.1. Child Factors

Child’s age and child’s sex: Each mother was asked about her child’s age and sex. Age was left as a continuous variable from the original parent study [20].

Infants and young children’s dietary diversity: This factor, described earlier in this paper, was measured using the WHO-developed CDD indicator for children 6–23 months of age. It is calculated based on the mother’s 24 h dietary recall of her child’s food consumption. Based on the WHO guidelines for infant and young children feeding practices, the food items were grouped into seven food groups/types: (1) grains, roots and tubers; (2) legumes and nuts; (3) dairy products; (4) flesh foods; (5) eggs; (6) vitamin A-rich fruits and vegetables; (7) other fruits and vegetables. The response options were “consumed” (1) and “not consumed” (0). The CDD score was calculated by adding the scores of all the food groups, making it a continuous variable ranging from 0 to 7.

#### 2.2.2. Maternal Factors

Maternal age: The mothers were asked their current age. This variable was left as a continuous measurement from the parent study.

Maternal education: The mothers were asked about their highest education level. The original response options were as follows: “no education”, “primary”, “secondary” and “higher” (from the original dataset). However, due to the frequency distribution of the education level, this variable was recoded as a binary variable of “no education” (0) and “primary and/or higher education” (1).

Hygiene of food preparation: The mothers were asked if they washed their hands before food preparation. The response options were either “yes” (1) or “no” (0). This variable was not recoded.

Woman’s empowerment: This factor, also known as the woman’s decision-making score, is defined as the number of decisions the woman participates in in her household. For the present study, we calculated the woman’s decision-making score by taking into account the DHS method of measuring the woman’s empowerment. Four indicators were used: (1) deciding on one’s health care; (2) deciding on how to spend one’s earnings; (3) deciding on making large household purchases and (4) deciding on visits to the family members or relatives. The woman’s empowerment variable was a total sum of the four indicators; the score ranged from 4 to 16, making it a continuous variable.

#### 2.2.3. Women’s Health Factors

Antenatal attendance: The mothers were asked about the number of antenatal visits they had during the pregnancy. This variable was left as a continuous measurement from the original dataset.

Vitamin A intake: The mothers were asked if they had received a vitamin A dose in the form of an ampoule, capsule or syrup in the previous 6 months. The response options were either “yes” (1) or “no” (0) (from the original dataset).

Current amenorrhea: The mothers were asked if they were currently amenorrhoeic. The response options were either “yes” (1) or “no” (0) (from the original dataset).

Anemia level: Trained staff used the HemoCue system to diagnose the anemia level. An informed voluntary consent form was read to the mothers before the administration of the test. Before collecting the blood, the finger was cleaned with an alcohol swab and air-dried. Then, the fingertip was pierced with a sterile and non-reusable retractable lancet. A drop of blood in a microcuvette was then introduced into a HemoCue photometer to measure the level of hemoglobin. This was recorded in the household questionnaire and communicated to the person tested. The original results were coded as “severe” (1), “moderate” (2), “mild” (3) and “not anemic” (4). Based on the frequency distribution of the original results, the results were recoded as “no anemia” (0) or “moderate, mild or severe anemia” (1) in the present study.

#### 2.2.4. Household Factors

Place of residence: The place of residence was coded as either “rural” (0) or “urban” (1). According to the USAID, “urban areas are classified as large cities (capital cities and cities with over 1 million population), small cities (population over 50,000), and towns (other urban areas), and all rural areas are assumed to be countryside” [20].

Wealth index: The wealth index is a composite measure of a household’s cumulative living standard. Based on the calculations, the original dataset responses were as follows: “poorest”, “poorer”, “middle”, “rich” and “richest”. This variable was recoded as “low” (0), “middle” (1) and “high” (2) income.

Number of living children: The mothers were asked about their total number of living children in the household. This number was left continuous, in its original form.

### 2.3. Data Analysis

The secondary analysis of the cross-sectional data was conducted using SPSS version 23.0. Descriptive statistics were used to calculate the sample means and standard deviations (SD) for continuous variables, while frequencies and percentages were calculated for categorial variables. Frequencies of food items were calculated by rural vs. urban areas and by regions. A chi-squared test was conducted to examine the association of the education level to the region. To compare the mean scores of CDD in relation to the region, an independent sample *t*-test was conducted [29]. A one-way ANOVA test was conducted to evaluate CDD score by different age groups [30]. Given that the CDD score, the outcome variable of interest, was continuous, a linear regression model was estimated to examine the association between the CDD score and the multiple factors involved at the community, household, maternal and child level. The association between the variables was considered to be statistically significant in cases where *p* ≤ 0.05 [31,32,33,34].

## 3. Results

### 3.1. Participants’ Characteristics

Table 1 reports the results of the descriptive analysis of the respondents. Our results indicate there was a significant association between the region and the education level; the mothers residing in rural areas were less likely to be educated than those in urban areas (X^(1)^ = 101.101, *p* < 0.05).

### 3.2. Children’s Dietary Diversity and Rural/Urban Patterns

Analyses revealed that grains were the most commonly consumed food group in both rural and urban areas (72.6% vs. 77.8%, respectively) (see Table 2). Our descriptive results also show that the regions with the highest consumption of legumes and nuts were Niamey (34%) and Agadez (29.4%) (see Table 3). Furthermore, vitamin A-rich fruits and vegetables consumption was relatively high in all the regions except for Maradi (18.8%) and Zinder (18.6%) (see Table 3). The CDD score was significantly higher in urban areas than in rural areas (*t* (1263) = −7.540 *p* < 0.05; *d* = 0.63).

The effect size for this analysis was found to exceed Cohen’s d convention for a medium effect (*d* = 0.5). There was no significant difference in CDD between the male and female children (*p* > 0.05) [34].

In addition, the results indicate that approximately 39% of children in each age group of our sample consumed foods from one food group and about 6% of children consumed foods from four different food groups (see Table 4). The one-way ANOVA test results indicate a significant difference in CDD scores between the different age categories of children (F (2, 1262 = 36.23), *p* ≤ 0.05), with children aged 19–23 months having a higher mean CDD score compared to other age groups (see Table 4).

### 3.3. Predictors of Children’s Dietary Diversity

Table 5 provides the results of the linear regression model for CDD. The estimated parameters were considered significant at *p* < 0.05. After controlling for all the other variables in the model, type of residence, child’s age in months, woman’s decision-making score, vitamin A intake and wealth index were significant indicators of CDD. For the children living in urban areas, the CDD score was 0.146 points higher than for those residing in rural areas. Furthermore, our results indicate that for each month of age, the CDD score increased by 0.24 (95% CI: 0.048, 0.076). The results show that the mothers who had received a vitamin A shot in the previous 6 months were most likely to have higher CDD (*p* < 0.05). In addition, a unit increase in the woman’s decision-making indicator was associated with a 0.05 increase in CDD (95% CI: 0.056, 0.088).

## 4. Discussion

This study examined CDD in Niger and the relationship between the maternal factors and CDD. The results of this study provide information that will aid public health practitioners and researchers in implementing effective nutritional health interventions to help address childhood malnutrition in Niger.

The results from the descriptive and regression analysis indicate that children residing in rural areas are more likely to have lower scores of dietary diversity, therefore becoming more susceptible to malnutrition. These findings are similar to those of other studies in that households in rural SSA are more likely to have lower dietary diversity scores than those in urban areas [35,36,37]. Furthermore, grains were the most commonly consumed food group among children, making our results consistent with findings of other studies from other countries [13,16,19,38]. These results are not surprising since approximately 75% of food consumption in Niger is composed of grains [39]. Additionally, the child’s age was significantly associated with a higher dietary diversity score. This finding is similar to that of previous studies conducted in other countries in SSA [8,9,39,40]. These results are not unexpected: as children grow, so does their ability to eat a variety of foods [3]. Moreover, children residing in wealthy households are more likely to have higher CDD scores than those from more disadvantaged households. This finding is similar to other studies [17,40].

The results of our study suggest a positive association between the maternal factors and CDD. After controlling for other variables, mothers receiving vitamin A was positively associated with CDD. Vitamin A deficiency affects approximately 40% of children 6–59 months of age in Niger [41]. Due to these high rates, the Nigerien government implemented the national vitamin A supplementation program by integrating vitamin A supplementation into the National Immunization Days (NIDs) for polio eradication in 1997. Additionally, in 1999, when National Micronutrient Days (NMDs) in Africa, a campaign independent from the NIDs, was implemented to mobilize caregivers to have their children receive vitamin A supplementation, the opportunity was also used to deliver vitamin A to postpartum mothers [42]. The NIDs and NMDs initiatives have been recognized and credited with increasing vitamin A uptake as well as the production and intake of foods rich in vitamin A [42]. The program’s success was due to its use of behavior change communication strategies [42] resulting in significant production and consumption of foods such as orange-fleshed sweet potatoes [43]. The supplementation program was moderately substantial among young children; however, it remained limited among mothers [42]. Our results show the impact of the vitamin A supplementation program on mothers since maternal vitamin A supplementation is associated with CDD. The program’s comprehensive nature, especially its integration of dietary behavior change education through social mobilization campaigns via mass media and key stakeholders, improved more than maternal postpartum supplementation, but also CDD, through its focus on improving nutrition [42]. It is imperative to stress the impact of the national vitamin A supplementation program on dietary diversity education. This implies that promoting and increasing maternal vitamin A supplementation was a paramount step in improving the nutritional dietary diversity of children.

Moreover, in our study, we found that an increased score in decision-making among mothers was associated with an increase in CDD. Although in their study [8] the gender empowerment variable was different (wife beating justified, yes or no), the researchers found that the women who did not believe in wife beating had children that had higher dietary diversity scores than those who did believe in wife beating. Furthermore, a related study in Ethiopia found that women of low status and high disempowerment scores were associated with poor health outcomes [44]. It is important to note that studies have found that poor maternal health is associated with poor child health [44,45,46]. Consequently, poor maternal health can contribute to poor dietary practices of children.

We believe this to be the first study that examined maternal decision-making score as it relates to CDD in Niger. Most studies on gender empowerment in SSA examine the role of empowerment as it relates to HIV/AIDS prevention and family planning [47,48]. Thus, more research is needed that examines the role of women’s decision-making and its impact on children’s dietary intake in SSA.

It is important to note the limitations of our study. First, the use of cross-sectional data limits our ability to examine trends over time. The use of a single 24 h dietary recall can hinder the ability to collect accurate dietary information about the respondent and their children over time [49,50]. Lastly, the 2012 Niger DHS data are the most recent available data. The 2017 data have not been released, and the 2021 DHS data collection is ongoing. Additionally, the parent study did not collect information on the dietary intake of the mothers; therefore, this study was not able to assess similarities and/or associations between the maternal dietary intake and that of their own children.

Despite limitations, our findings have implications for nutritional studies and interventions globally. Future studies aimed to reduce malnutrition rates should no longer limit their focus to solely researching dietary intake. We recommend that studies take into consideration policies and/or programs that impact healthy eating for children and their families. With regard to nutritional interventions, the results of this study stress the importance of considering ecological factors influencing nutritional outcomes beyond individual behaviors. The BEM stresses the bidirectional influence of factors at the social/cultural, community, local and individual levels [23,24]. Thus, food production in Niger alone cannot make a significant impact on dietary practices unless it is accompanied with women’s empowerment and culturally appropriate nutrition education [9,51].

## 5. Conclusions

Our results demonstrate that the majority of children in Niger 6–23 months of age have low dietary diversity. Maternal factors such as vitamin A supplementation and decision-making score were significant predictors of dietary diversity. Hence, health promotion programs should follow the precedent of the vitamin A program by integrating and utilizing the existing programs and initiatives as avenues to emphasize the importance of diverse diets for women and children through health education. Furthermore, nutritional education programs should take into account the availability of food and the eating behavior patterns of the population. Future research aimed to reduce malnutrition should examine successful strategies to implement evidence-based interventions that improve dietary diversity while taking into account women’s empowerment.

## Figures and Tables

**Table 1:** Characteristics of the respondents (*n* = 1265).

Demographic Variables	Mean ± SD
Child’s age in months	13.45 ± 5.08
Maternal age	28.45 ± 6.58
Number of living children	3.93 ± 2.19
Number of antenatal visits during pregnancy	3.22 ± 5.57
Woman’s decision-making score	7.7 ± 2.21
Child’s sex	
Male	49.2
Female	50.8
Maternal education	
No education	80.7
Primary and/or higher	19.3
Maternal anemia level	
No anemia	58
Moderate-to-severe	42
Hygiene level before food preparation	
No	15.3
Yes	84.7
Wealth Index	
Poor	33.4
Middle	19.8
Rich	46.8
Region	
Urban	8.5
Rural	91.5

**Table 2:** Percentage of children consuming a specific food group by rural and urban areas.

Food Group	Rural	Urban
Grains	72.6	77.8
Meat	11.6	34.3
Dairy	14.4	26.9
Legumes and nuts	12.4	30.6
Eggs	4.2	12
Vitamin A-rich fruits and vegetables	26.4	38
Other fruits	4.4	24.1

**Table 3:** Percentage distribution of food groups by region.

Regions (%)								
Food Type	Agadez	Diffa	Dosso	Maradi	Tahoua	Tillaberi	Zinder	Niamey
Grains	60.8	45.2	82.9	76	88.7	81.2	61.5	86.2
Meat	23.5	17.8	10.7	10.8	12.4	14.7	8.3	39.4
Dairy	21.6	5.5	1.1	9.2	8.6	5.3	0.6	10.6
Legumes and nuts	29.4	24.7	11.8	11.6	15.1	6.5	10.3	34.0
Eggs	11.8	5.5	1.1	2	3.8	9.4	5.1	12.8
Vitamin A-rich fruits and vegetables	31.4	24.7	34.2	18.8	39.2	31.2	18.6	43.6
Other fruits	3.9	5.5	1.6	6	9.7	1.8	3.2	27.7

**Table 4:** Children’s dietary diversity (CDD) by age category.

Food Groups	6–12 Months	13–18 Months	19–23 Months
0	31.50%	13.50%	6.90%
1	38.90%	37.10%	38.50%
2	15.60%	25.70%	28.50%
3	8.00%	11.90%	15.40%
4	2.90%	7.60%	6.20%
5	2.60%	2.90%	3.50%
6	0.50%	1.20%	1.20%
7	0.00%	0.20%	0.00%
CDD, mean	1.22 ± 1.24	1.77 ± 1.33	1.90 ± 1.24
CDD, median	1	1	2
CDD, min	0	0	0
CDD, max	6	7	6

**Table 5:** Results of the linear regression analysis of CDD in Niger.

Variable	B	95% CI	*p*-Value
Type of residence			
Rural	reference		
Urban	0.146	(0.421, 0.951)	0.000
Child’s sex			
Male	reference		
Female	−0.012	(−0.168, 0.103)	0.639
Child’s age	0.241	(0.048, 0.076)	0.000
Mother’s age	0.024	(−0.011, 0.021)	0.311
Maternal education			
No education	reference		
Primary and/or higher	0.040	(−0.019, 0.364)	0.078
Decision-making score	0.052	(0.056, 0.088)	0.009
Wash hands before food preparation			
No	reference		
Yes	0.035	(−0.065, 0.318)	0.183
Vitamin A in the previous 6 months			
No	reference		
Yes	0.080	(0.074, 0.358)	0.000
Anemia level			
No anemia	reference		
Moderate-to-severe	−0.004	(−0.149, 0.129)	0.885
Currently amenorrhoeic			
No	reference		
Yes	−0.040	(−0.252, 0.044)	0.165
Number of antenatal visits	−0.003	(−0.013, 0.012)	0.916
Number of living children	−0.001	(−0.049, 0.047)	0.973
Wealth Index			
Low	reference		
Middle	−0.008	(−0.167, 0.218)	0.797
High	0.097	(0.087, 0.423)	0.033

Notes. R^2^ = 0.33, *p ≤* 0.05. B, B-coefficients.

## Data Availability

The data presented in this study are available at https://dhsprogram.com/data/dataset/Niger_Standard-DHS_2012.cfm?flag=0, accessed on 15 April 2016.
